# Supplementary material for: Recovery of sweet taste preference in adult rats following bilateral chorda tympani nerve transection
Source: PeerJ. 2022 Nov 25;10:e14455. doi: 10.7717/peerj.14455 (PMC9703994; doi:10.7717/peerj.14455)
Supplement: Supplemental Information 1 [file peerj-10-14455-s001.zip › PeerJ Supplementary CTX/Supplementary data.pptx]

## Slide 1
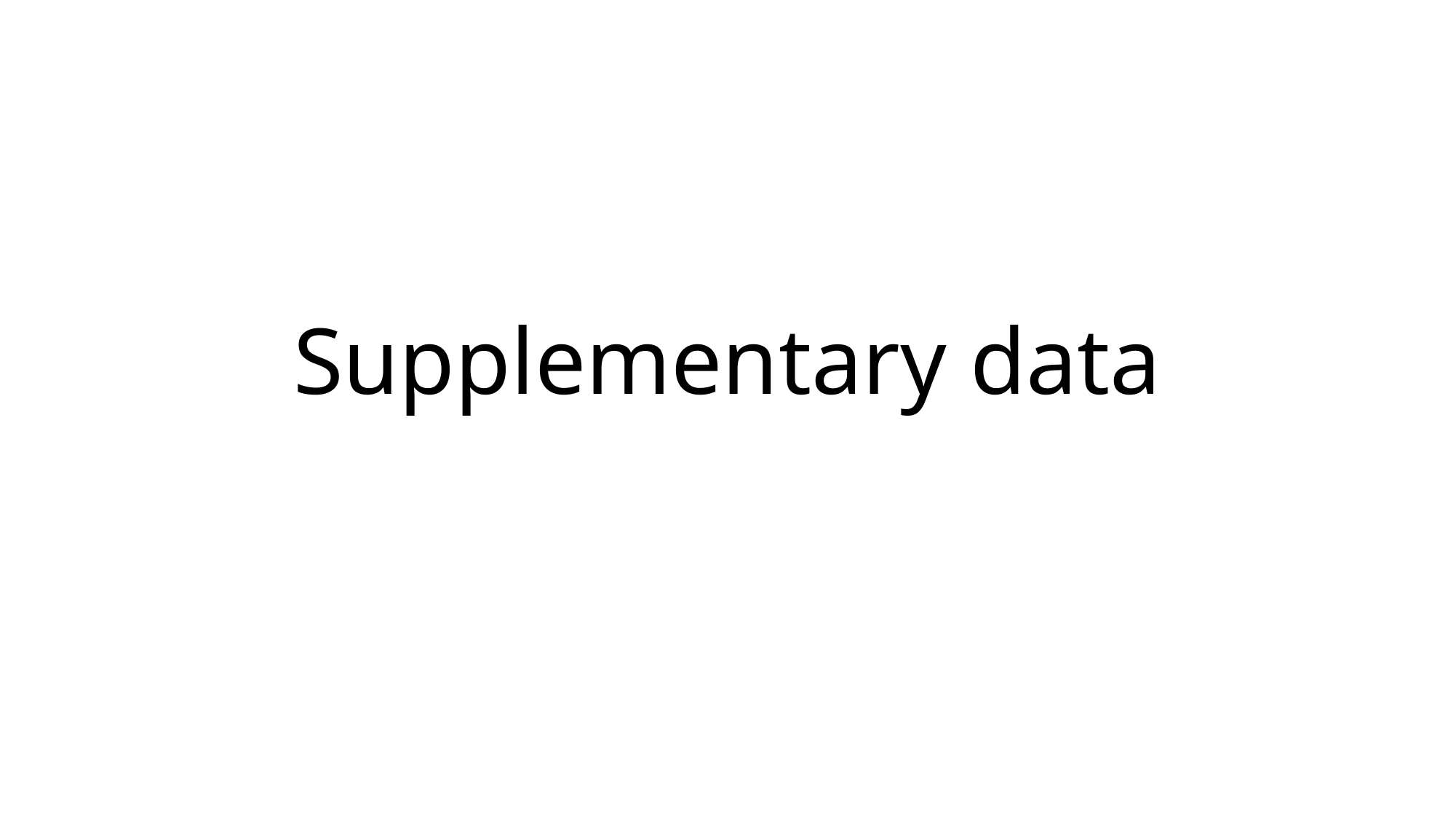

# Supplementary data

## Slide 2
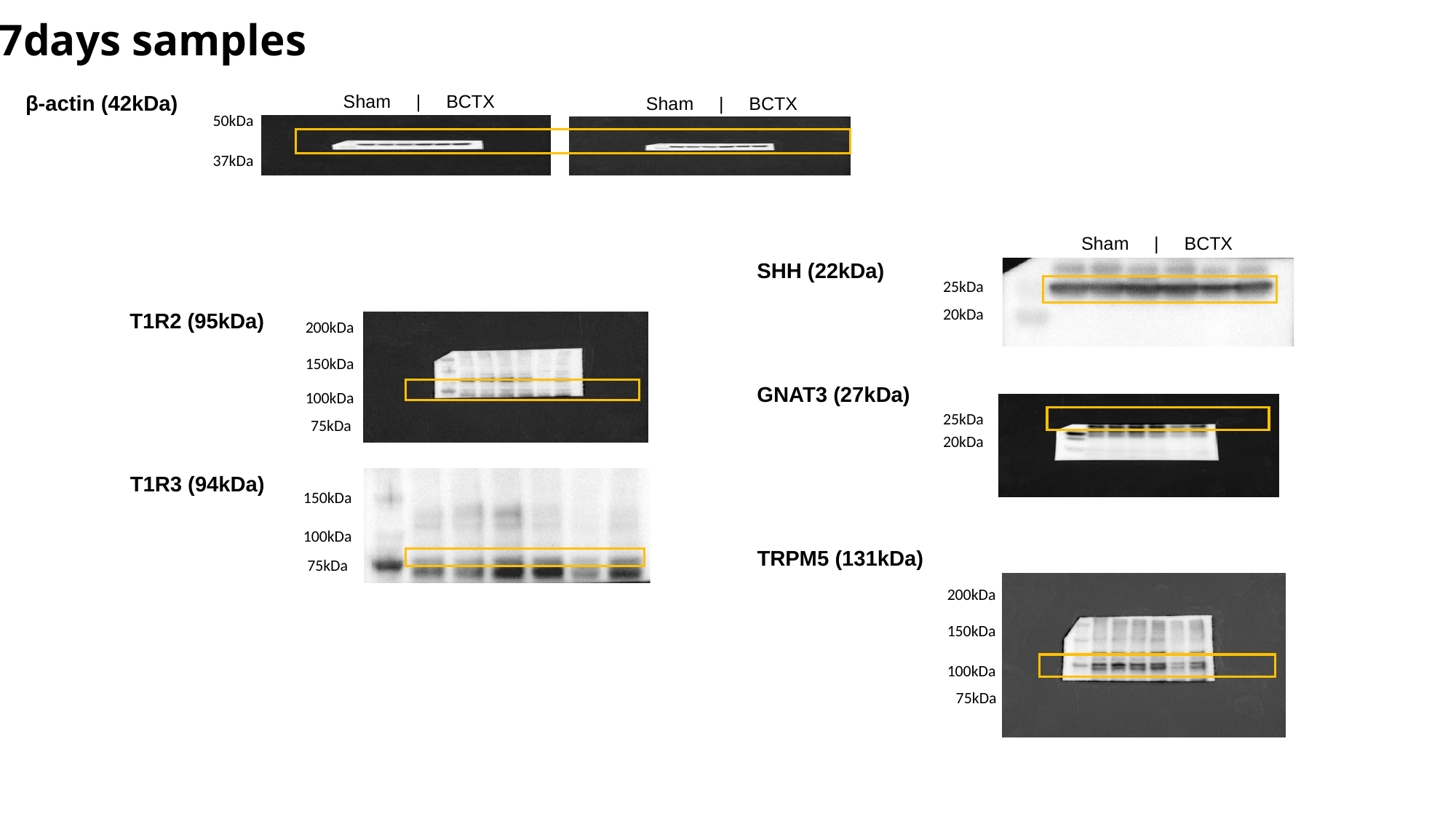

7days samples
β-actin (42kDa)
Sham | BCTX
Sham | BCTX
50kDa
37kDa
Sham | BCTX
SHH (22kDa)
25kDa
20kDa
T1R2 (95kDa)
200kDa
150kDa
GNAT3 (27kDa)
100kDa
25kDa
75kDa
20kDa
T1R3 (94kDa)
150kDa
100kDa
TRPM5 (131kDa)
75kDa
200kDa
150kDa
100kDa
75kDa

## Slide 3
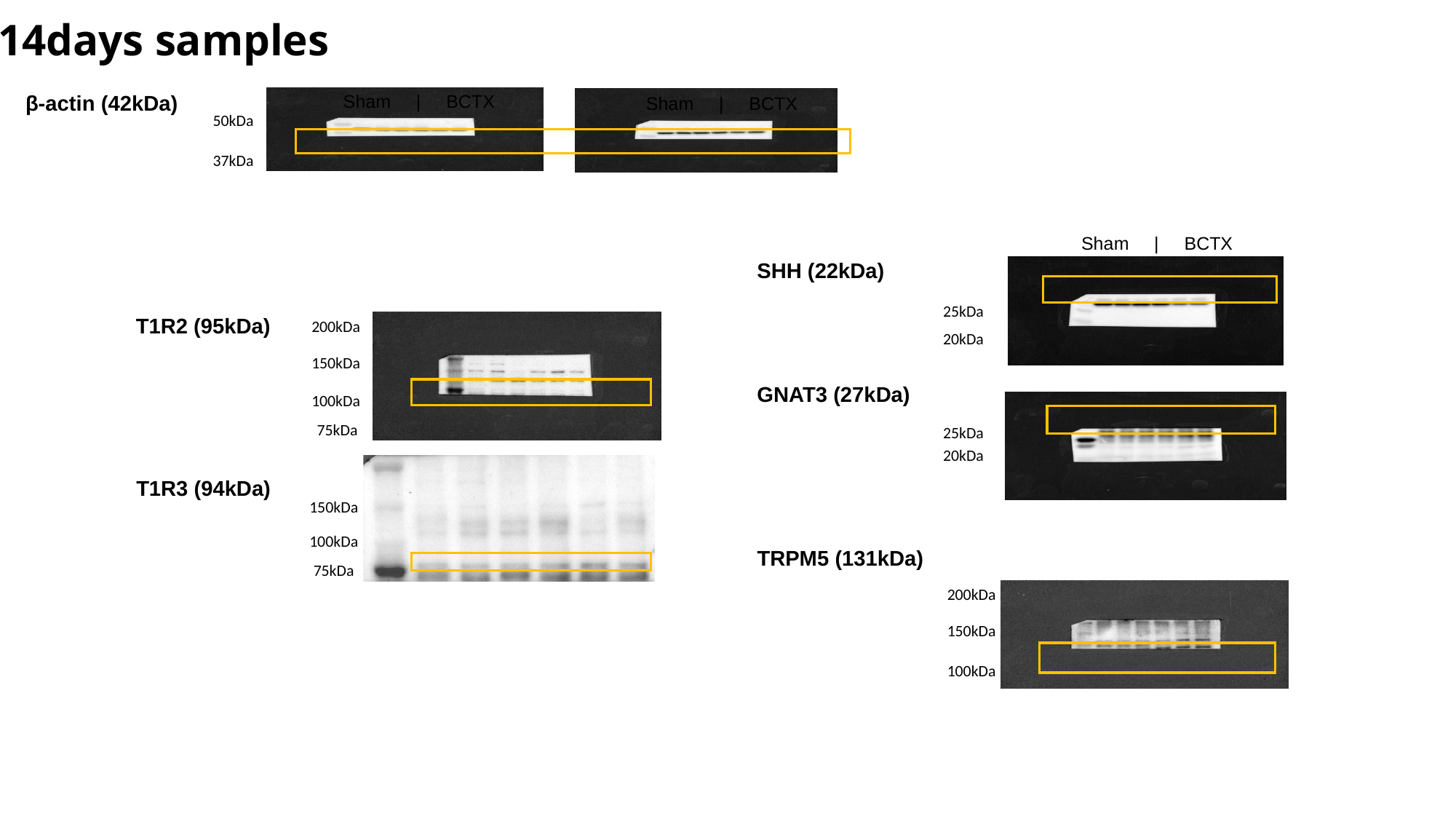

14days samples
β-actin (42kDa)
Sham | BCTX
Sham | BCTX
50kDa
37kDa
Sham | BCTX
SHH (22kDa)
25kDa
T1R2 (95kDa)
200kDa
20kDa
150kDa
GNAT3 (27kDa)
100kDa
75kDa
25kDa
20kDa
T1R3 (94kDa)
150kDa
100kDa
TRPM5 (131kDa)
75kDa
200kDa
150kDa
100kDa

## Slide 4
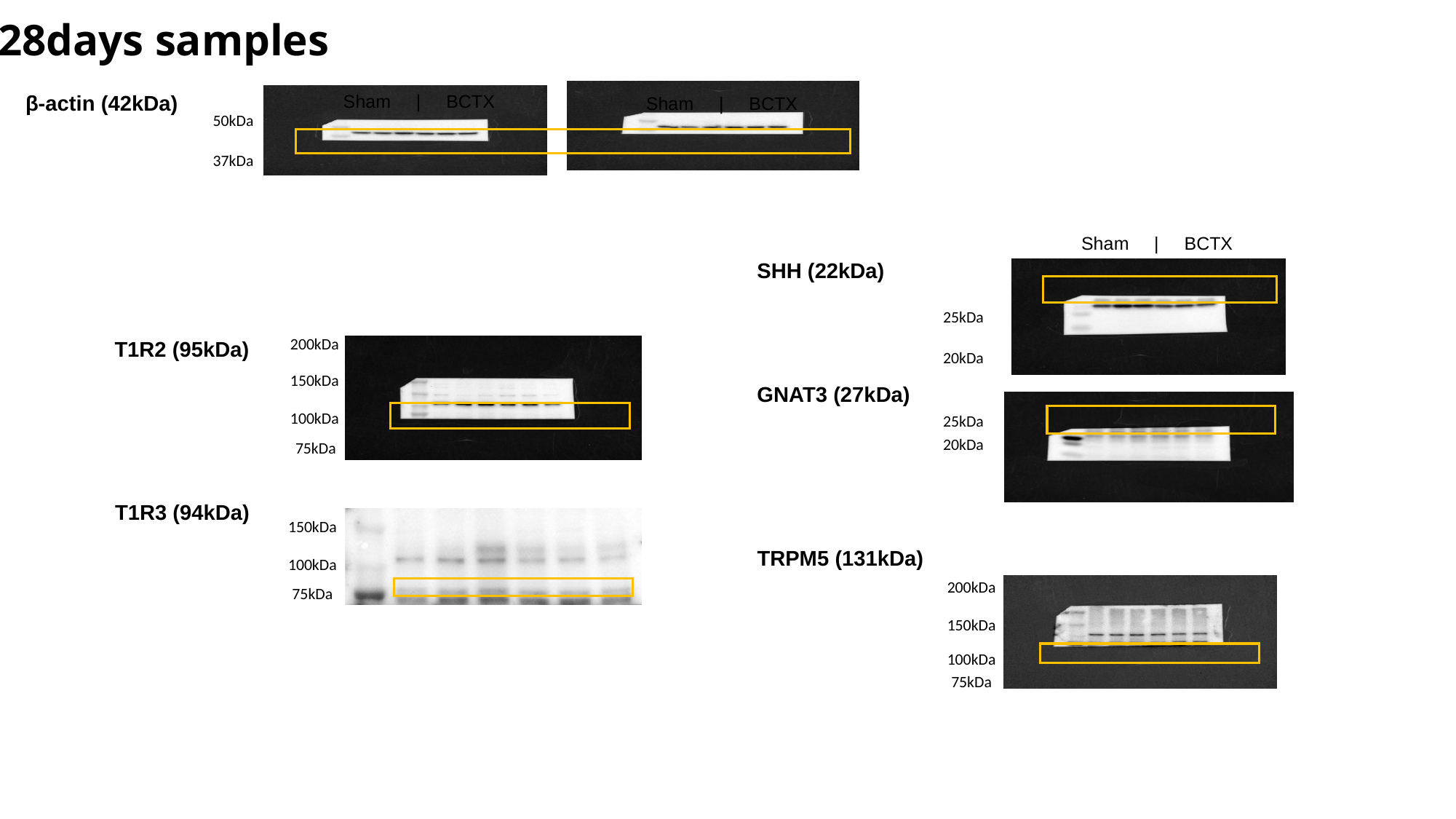

28days samples
β-actin (42kDa)
Sham | BCTX
Sham | BCTX
50kDa
37kDa
Sham | BCTX
SHH (22kDa)
25kDa
200kDa
T1R2 (95kDa)
20kDa
150kDa
GNAT3 (27kDa)
100kDa
25kDa
20kDa
75kDa
T1R3 (94kDa)
150kDa
TRPM5 (131kDa)
100kDa
200kDa
75kDa
150kDa
100kDa
75kDa
